# Bad Colds

**Published:** 1857-08

**Authors:** 


### BAD COLDS.

VERY many of the most hopeless forms of consumption are the indirect result of colds taken in the summer time, more perhaps, in the six weeks embracing the fourth of July, than any other three months in the year. The reason is, that a very little exercise, especially if unusual, causes large perspiration, and from that, as well as from that bodily debility which is always present in midsummer, the system has but feeble powers of resistance, and is to a great extent helpless against the onsets of disease, especially against cold. While thus debilitated and perspiring, if the person is at rest but a few minutes in a draft of air; or, if having been in a shower of rain, he is compelled to remain still, from being in a carriage, or from any other reason, a severe cold is almost inevitable. We have a long list of women, (especially at the age of about eighteen, far more liable at the critical season) whom no skill could save from the effects of causes just pointed out.

The same amount of "a wetting" in mid-winter, would not have caused any special inconvenience, because there is something in cold weather which impels to exercise, and this keeps up a vigorous circulation of the blood, a condition in which it is impossible to take cold; and this circulation dries the clothing by the heat which it generates.

The rule, applicable universally is, whenever you get wet, especially in summer time, set yourself instantly in motion and keep it up until you can change your clothing. The best way of doing which, is to take a hearty drink of "something," or red pepper tea or other kind, the hotter the better, the moment you enter the house; and while undressing have the dry clothing made hot by the fire, lose but a single minute in wiping yourself dry. Dress rapidly, "take another drink," eat a meal, or exercise about the room until completely warmed.

To show how much out-door exposure in winter-time, may be encountered with impunity, it may be stated, that in March last, a party of Indians at Spirit Lake, Iowa, took Mrs. Marble from the residence of her husband. Her warm clothing was divided among the squaws, leaving her very thinly clad. In this condition, with a deep snow on the ground, she was compelled to march, and carry on her back a bag containing fifty pounds of shot, on which was placed an Indian child three years old. At times, when suffering from hunger, she would almost faint with fatigue, when the savages would point their guns to her head, and threaten instant death unless she proceeded. She was glad to get the bones which her captors threw away from their meals, and often ate the wing-feathers, plucked from ducks which had been shot. At the end of many days she arrived at a station in good health, and was redeemed by some traders.

The ABBE LEMMENIER, as a witness in an important trial says, that of sixty thousand pilgrims who passed the night in the snow, with their heads uncovered in a freezing fog, he did not know of a single person who took cold. Persons who are immersed, religiously, in open streams in mid-winter, even when the ice has been broken. seldom take cold; but jumping into a river to bathe, in the summer time, while a little heated, has often induced fatal diseases. Beware then, of draughts of air, wet clothing, and damp rooms in warm weather.

